# Alcohol and psychoactive substance use in a cohort of children followed by child protection in France

**DOI:** 10.3389/fpsyt.2023.1180292

**Published:** 2023-10-25

**Authors:** Aziz Essadek, Maeva Musso, Adèle Assous, Frédéric Widart, Joris Mathieu, Marion Robin, Gérard Shadili

**Affiliations:** ^1^Université de Lorraine, INTERPSY, Nancy, France; ^2^Department of Psychiatry, Hopital Saint-Maurice, Paris, France; ^3^University of Paris, CRPMS, Paris, France; ^4^Department of Psychology, University of Liège, Liège, Belgium; ^5^Centre Spécialisé de l’Obésité (CSO) et Service d’Endocrinologie, Diabétologie et Nutrition (EDN), CHRU-Nancy, Vandoeuvre-lès-Nancy, France; ^6^Department of Adolescent and Young Adult Psychiatry, Institut Mutualiste Montsouris, Paris, France; ^7^INSERM U1178, Team PsyDev, Villejuif, Paris, France

**Keywords:** substance use, child protection, mental health, youth & adolescence, maltreatment

## Abstract

**Background and aim:**

Many studies have investigated the association between maltreatment and substance use in adulthood.

In this study, we sought to explore the association between substance use during adolescence, diverse forms of child maltreatment, and psychological symptoms within a cohort of individuals under the purview of child protection services in France.

**Method:**

The dataset was culled from a retrospective, population-based study encompassing minors and young adults aged 0 to 21, who were under the care of child protection services. Specifically, we conducted a comparative analysis between minors exhibiting substance use (N = 72) and those without such use (*N* = 776).

**Result:**

The odds ratios predominantly illuminated a significant correlation between Substance Use and the manifestation of self-destructive behavior (OR = 4.35; CI 2.02–9.59), as well as aggressive behavior (OR = 5.75; CI 2.87–11.84). Univariate analysis also hinted at an association between SUD and suicidal ideation (OR = 3.52; CI 2.1–5.90).

**Conclusion:**

Children in France who are in the care of child protection services and who use psychoactive substances are at greater risk of dropping out of school and of having other psychological symptoms. It is important that the public authorities take account of these results in order to adjust the care given to these minors, who often do not receive psychological support.

## 1. Introduction

In France, alcohol and substance use are major public health problems. In the general population, initial consumption appears around the age of 13.3 years for boys and 13.5 years for girls (1). It is estimated that 7% of deaths the population aged 15 and over are related to alcohol consumption (2). Alcohol consumption starts very early, and is often associated with the use of other substances. Indeed, nearly 10% of 17 year olds are regular multiple users of alcohol, tobacco or cannabis (3). This issue appears to have an international dimension, as a recent Australian study showed that alcohol was the main cause of death and hospitalization among young Australians (15–24 years) (4). At this age, substance use is often associated with partying (5), youth justify their consumption by the sensations it provides, the desire to get intoxicated, or to facilitate integration into a group (3). However, several studies have highlighted the risk of correlation with other psychopathologies (6) and a risk of future mental health impairment (7).

A key point highlighted in the literature is that alcohol or substance use in adolescence and adulthood is significantly associated with maltreatment and negative childhood experiences (7). More globally, children’s experiences and living conditions, from pregnancy onwards (8) have a major influence on the future mental health of victims. Indeed, the World Health Organization (WHO) claims that one in four adults worldwide report having experienced physical violence in childhood (9). A WHO study (10) reports that childhood adversity accounts for 29.8% of all disorders in all countries. In France, in 2015, between 17.6 and 22.0% of women, and between 12.9 and 18.0% of men reported having experienced at least one form of abuse during childhood (11), which may have major consequences in terms of public health. Numerous studies have highlighted the consequences of child maltreatment on the mental health of individuals. Moreover, numerous studies have pointed out the bidirectional relationship between mental health and SUD (12). Alcohol consumption can significantly trigger and exacerbate symptoms of internalization, and conversely, symptoms of internalization increase a person’s vulnerability to developing SUD (13). Therefore, these adolescents who exhibit vulnerabilities appear to be at a higher risk of developing psychological symptoms associated with SUD. While these factors have been identified in the general population, we have less evidence for children in child protection care, despite overexposure to maltreatment, but also to mental health disorders (14). Additionally, studies on substance use among minors in child protection services are less existing, especially in France. It therefore seems important to us to focus on this population, which is exposed to multiple forms of abuse (15). Indeed, a cumulative phenomenon of exposure to abuse during childhood seems to amplify the risk of developing a psychological pathology (16–18). Besides, this cumulative aspect has a negative impact on the reduction of psychological symptoms over time (19).

A recent study underlined the complexity of estimating substance use among minors in child welfare services, which depends on the way in which it is measured and the age of the sample (20). Thus, rates ranging from 20% (21) to 50% (22) have been identified. It is essential to take this issue into account, as substance use is strongly associated with delinquency (23) and homelessness (24) in child protection populations, as reported by Bransard et al. (14), who point out that 25% of homeless people and 20% of adults in prison in France were previously child protection beneficiaries.

Considering maltreatment and its specificity as a risk factor for mental health disorders, and in particular substance and alcohol abuse, implies modifying care to adapt it to this population by favoring trauma-focused approaches to combat the long-term impact of the abuse (25). While many studies highlight the impact of maltreatment on the risk of illicit substance use and mental health deterioration, few studies have focused specifically on populations of maltreated children in the care of social services. The primary aim of the study was to ascertain whether Substance Use Disorders (SUD) among these minors varied based on the type of maltreatment experienced. The second objective was to examine the association between SUD and the presence of other psychological symptoms.

## 2. Methods

The present study relies on an analysis of data collected as part of the population-based study on children and young adults accompanied by the Essonne departmental council in the context of child protection. This is a retrospective cross-sectional study. The aim of this population-based study is to highlight the characteristics of maltreatment of minors in the Aide Sociale à l’Enfance (ASE) and to examine the psychological disorders of minors and young adults. It was developed from the Canadian incidence study (26). To conduct this population-based study, an email was sent to all social workers in charge of measures, in which a link was provided to allow them to complete the tool. We used the LimeSurvey software, so that the survey could be completed online. Professionals had between June 2020 and November 2020 to complete the survey. In total, 1,557 records were partially completed. 242 files were discarded because of too many missing answers. The result is a set of 193 variables containing information on 1,315 completed files, i.e., 19.185% of minors in care in the whole department.

As part of this research, we worked on the following variables Socio-demographic data: age, sex, duration of care, legal status of the child, interaction of the child with the parents, schooling, MDPH recognition (departmental house for the disabled people) and psychological care of the minor.

### Data related to maltreatment

The data collected in the files are those that were included in the reason for care by the child protection services. These are physical abuse, sexual abuse, neglect, exposure to domestic violence, and psychological abuse. In terms of the timeline of events, the maltreatment comes first. Indeed, they prompted the need for intervention by child protection services. The symptoms were assessed at a later stage.

### Data on psychological disorders

From the administrative files, social workers were asked to record psychological symptoms in children: depression, anxiety, suicidal ideation, self-destructive behavior, Attention Deficit Hyperactivity Disorder (ADHD), aggressive behavior. This research was recorded in the Université de Lorraine’s research register.

In this research, we focused on young individuals who engage in psychoactive substance consumption. Substance consumption is defined as a daily consumption of cigarettes and/or cannabis, as well as regular alcohol consumption. A first group of SUD youth was formed (N = 72) was compared to a group without substance use (*N* = 776). To do so, we excluded all children with an age of 11 years or younger because in our cohort, no child under the age of 11 presented cigarette, cannabis, and/or alcohol consumption.

## Statistical analyses

Data analyses were performed using R software (version 4.0.2) and the level of significance was set at 0.05. In a first step, we performed descriptive analyses. The distribution of continuous data was estimated using the Anderson-Darling test. As the continuous variables are all non-parametric, they were described using medians and interquartile ranges (IQR). Categorical variables were reported in terms of number (n) and percentage (%).

We opted to perform several multivariable binary logistic regression models to explore the association of SUD. The results are presented in terms of odds ratios (OR) with 95% confidence intervals (95% CI). First, we explored the association of CPSA with sociodemographic variables (Model 1). Second, we investigated the association of CPSA with all childhood maltreatment variables (Model 2). Third, we examined the association between CPSA and the set of psychological symptoms variables (Model 3). Fourth, we included both sociodemographic and maltreatment variables (Model 4). Fifth, we included both sociodemographic and psychological symptoms variables (Model 5). Finally, we integrated sociodemographic, maltreatment, and psychological symptoms variables into the model (Model 6). Variation inflation factors (VIF <10) were calculated to check for non-correlation and multicollinearity (27). All the eigenvalues of the VIF were less than 10. With these results, multicollinearity has been ruled out as a concern. Finally, we calculated the Nagelkerke and McFadden *R^2^* for each model.

## Results

In the entire cohort, a total of 72 adolescents were afflicted by SUD. The median age for this subgroup of minors was 17 years [15–19], with 41 (56.9%) being male. The predominant legal status among these adolescents with SUD was categorized as parental authority, encompassing 38 cases (52.8%). Interactions between parents and offspring were predominantly characterized as “frequent,” representing 25 instances (40%). A substantial portion of this cohort of minors, approximately 47.2%, is found to be disengaged from formal education. Among these individuals, 9 cases (12.5%) had received formal recognition for having a recognized disability. Notably, a considerable subset of these adolescents, totaling 30 cases (41.7%), were concurrently receiving psychological support. All socio-demographic variables are described in Table 1.

**Table 1:** Characteristics of the SUD^a^ and others.

	SUD (72)	Other (776)	All (848)
	Median [IQR] / *n* (%)	Median [IQR] / *n* (%)	Median [IQR] / *n* (%)
Socio-demographic variables			
Age	17 [15–19]	16 [12–20]	16 [4]
Sex			
Female	31 (43.05)	339 (43.68)	370 (43.63)
Male	41 (56.94)	437 (56.31)	478 (56.37)
Status of the child			
Parental authority	38 (52.78)	451 (58.12)	489 (57.67)
Adult CJM^b^	21 (29.16)	175 (22.55)	196 (23.11)
Delegation of authority	6 (8.33)	37 (4.77)	43 (5.07)
Pupil of the state	1 (1.38)	21 (2.71)	22 (2.59)
Unaccompanied minors awaiting status	5 (6.94)	60 (7.73)	65 (7.67)
Other	1 (1.38)	32 (4.12)	33 (3.89)
Parent–child interaction			
Permanent	5 (6.94)	121 (15.59)	126 (14.86)
Often	25 (34.72)	243 (31.31)	268 (31.6)
Rarely	20 (27.78)	166 (21.39)	186 (21.93)
Never	22 (30.56)	246 (31.70)	268 (31.6)
Education			
Yes	34 (52.78)	676 (87.11)	714 (84.2)
No	38 (47.22)	100 (12.89)	134 (15.8)
Recognition MDPH (Departmental house for disabled people)			
Yes	9 (12.5)	170 (21.91)	179 (21.11)
No	63 (87.5)	606 (78.09)	669 (78.89)
Psychological support			
Yes	30 (41.67)	360 (46.39)	390 (45.99)
No	42 (58.33)	416 (53.61)	458 (54.01)
Child maltreatment			
Physical Abuse			
Yes	38 (52.78)	332 (42.78)	366 (43.16)
No	34 (47.22)	444 (57.22)	482 (56.84)
Sexual abuse			
Yes	16 (22.22)	138 (17.78)	154 (18.16)
No	56 (77.78)	638 (82.22)	694 (81.84)
Neglect			
Yes	47 (65.28)	527 (67.91)	574 (67.69)
No	25 (34.72)	249 (32.09)	274 (32.31)
Psychological violence			
Yes	39 (54.16)	444 (57.22)	483 (56.96)
No	33 (45.83)	332 (42.78)	365 (43.04)
Domestic violence			
Yes	27 (37.5)	349 (44.97)	376 (44.34)
No	45 (62.5)	427 (55.03)	472 (55.66)
Psychological symptoms			
Depression			
Yes	40 (55.56)	354 (45.62)	394 (46.46)
No	32 (44.44)	422 (54.38)	454 (53.54)
Anxiety			
Yes	54 (75)	557 (71.78)	611 (72.05)
No	18 (25)	219 (28.22)	237 (28.95)
Suicidal Ideation			
Yes	27 (37.5)	113 (14.56)	140 (16.51)
No	45 (62.5)	663 (85.45)	708 (83.49)
Self-destructive behavior			
Yes	44 (61.11)	164 (21.13)	208 (24.53)
No	28 (38.89)	612 (78.86)	640 (75.47)
ADHD^4^			
Yes	21 (29.17)	221 (28.48)	242 (28.54)
No	51 (70.83)	555 (71.52)	606 (71.46)
Attachment disorder			
Yes	42 (58.33)	396 (51.03)	438 (51.65)
No	30 (41.67)	380 (48.97)	410 (48.35)
Aggressive Behavior			
Yes	49 (68.05)	202 (26.03)	251 (29.6)
No	23 (31.94)	574 (73.97)	597 (70.4)

a Substance use disorders.

b Contrat jeune majeur (Young Adult Contract: This refers to a type of legal agreement or program that provides support and assistance to young individuals who are transitioning from the status of minors to adults in the child protection context).

### Univariable analysis

Table 2 presents the odds ratios estimating the association for SUD (Substance Use Disorder) with different variables. SUD was associated with higher age [1.21; CI (1.08–1.36); *p* < 0.001], infrequent parental interactions [2.91; CI (1.14–8.96); *p* = 0.037], suicidal ideation [3.52 (2.10–5.9); *p* < 0.001], Self-destructive behavior [5.86; CI (3.54–9.71); *p* < 0.001], and Aggressive Behavior [6.05; CI (3.6–10.19); *p* < 0.001]. Conversely, being enrolled in school appeared to reduce the risk of SUD [0.16; CI (0.09–0.27); *p* < 0.001]. Our results highlight that a form of maltreatment is significantly associated with SUD.

**Table 2:** Univariable binary logistic regression models.

	OR 95% CI	*p* value
Age	**1.21 (1.08–1.36**)	**<0.001**
Sex (Male)	1.02 (0.63–1.67)	1
Status of the child		
Parental authority (reference)	–	
Adult CJM^1^	1.42 (0.8–2.47)	0.216
Delegation of authority	1.92 (0.69–4.55)	0.165
Pupil of the state	0.56 (0.03–2.82)	0.582
Unaccompanied minors awaiting status	0.99 (0.33–1.40)	0.982
Other	0.37 (0.02–1.80)	0.335
Parent–child interaction	–	
Permanent (reference)	–	
Often	2.49 (1.01–7.52)	0.069
Rarely	**2.91 (1.14–8.96)**	**0.037**
Never	2.86 (0.53–6.59)	0.128
Education (Yes)	**0.16 (0.09–0.27)**	**<0.001**
Recognition MDPH (Yes)	0.51 (0.25–1.04)	0.07
Psychological support (Yes)	0.82 (0.51–1.34)	0.46
Physical Abuse (Yes)	1.20 (0.74–1.94)	0.53
Sexual abuse (Yes)	1.32 (0.73–2.37)	0.34
Neglect (Yes)	0.89 (0.53–1.47)	0.693
Psychological violence (Yes)	0.88 (0.54–1.43)	0.62
Domestic violence (Yes)	0.73 (0.45–1.21)	0.26
Depression (Yes)	1.49 (0.92–2.42)	0.11
Anxiety (Yes)	1.17 (0.68–2.06)	0.68
Suicidal Ideation (Yes)	**3.52 (2.10–5.90)**	**<0.001**
Self-destructive behavior (Yes)	**5.86 (3.54–9.71)**	**<0.001**
ADHD (Yes)	1.03 (0.61–1.76)	0.89
Attachment disorder (Yes)	1.34 (0.82–2.19)	0.27
Aggressive Behavior (Yes)	**6.05 (3.6–10.19)**	**<0.001**

^1^Contrat jeune majeur (Young Adult Contract: This refers to a type of legal agreement or program that provides support and assistance to young individuals who are transitioning from the status of minors to adults in the child protection context).

### Analysis of models

Table 3 presents the results of multivariable binary logistic regression models applied to analyze Substance Use Disorder (SUD). Elevated age and disengagement from formal education demonstrated persistent associations with SUD across the models. Additionally, it can be highlighted that self-destructive behavior and aggressive behavior also exhibited consistent associations across all models. However, the association with frequent parental interactions was significant only within Models 5 and 6. Moreover, the status of being classified as an emerging adult exhibited an association with SUD only within Models 1 and 4.

**Table 3:** Comparison of logistic regression models to explain SUD.

	Model 1 N = 848	Model 2 N = 848	Model 3 N = 848	Model 4 N = 848	Model 5 N = 848	Model 6 N = 848
	OR 95% CI	OR 95% CI	OR 95% CI	OR 95% CI	OR 95% CI	OR 95% CI
Age	**1.23 (1.01–1.52**)	–	–	**1.24 (1.01–1.53)**	**1.40 (1.11–1.79)**	**1.39 (1.09–1.79)**
Sex (Male)	1.04 (0.61–1.78)	–	–	1.05 (0.60–1.88)	1.08 (0.59–2.02)	0.97 (0.5–1.89)
Status of the child		–	–			
Parental authority (reference)	-	–	–	–	–	–
Adult CJM^1^	**0.37 (0.14–0.94)**	–	–	**0.36 (0.14–0.92)**	0.51 (0.18–1.41)	0.5 (0.17–1.40)
Delegation of authority	2.37 (0.77–6.50)	–	–	2.21 (0.71–6.15)	2.99 (0.85–9.66)	3.03 (0.84–9.92)
Pupil of the state	0.51 (0.02–3.24)	–	–	0.49 (0.02–3.15)	1.36 (0.06–9.98)	1.17 (0.05–8.92)
Unaccompanied minors awaiting status	0.38 (0.10–1.23)	–	–	0.33 (0.09–2.32)	0.70 (0.17–2.54)	0.6 (0.14–2.28)
Other	0.41 (0.02–2.17)	–	–	0.44 (0.98–8.19)	0.44 (0.02–2.81)	0.45 (0.02–3)
Parent–child interaction	-	–	–	–	–	–
Permanent (reference)	-	–	–	–	–	–
Often	2.51 (0.96–7.91)	–	–	2.58 (0.99–8.19)	**3.73 (1.31–12.64)**	**3.57 (1.23–12.24)**
Rarely	2.21 (0.80–7.21)	–	–	2.34 (0.82–7.75)	2.78 (0.91–9.77)	3.08 (0.98–11.14)
Never	1.57 (0.53–5.32)	–	–	1.60 (0.54–5.50)	2 (0.62–7.41)	2 (0.6–7.55)
Education (Yes)	**0.15 (0.08–0.27)**	–	–	**0.15 (0.08–0.27)**	**0.24 (0.12–0.46)**	**0.23 (0.12–0.45)**
Recognition MDPH (Yes)	0.45 (0.19–0.97)	–	–	0.47 (0.20–1.02)	**0.37 (0.14–0.91)**	**0.39 (0.14–0.96)**
Psychological support (Yes)	1.12 (0.63–1.95)	–	–	1.11 (0.62–1.97)	0.91 (0.47–1.72)	0.95 (0.49–1.82)
Physical Abuse (Yes)	–	1.43 (0.79–2.60)	–	1.28 (0.67–2.47)	–	0.97 (0.46–2.01)
Sexual abuse (Yes)	–	1.41 (0.73–2.59)	–	1.29 (0.62–2.59)	–	1.07 (0.46–2.37)
Neglect (Yes)	–	0.96 (0.54–1.74)	–	1.02 (0.54–1.95)	–	1.11 (0.55–2.26)
Psychological violence (Yes)	–	0.81 (0.44–1.5)	–	0.74 (0.37–1.46)	–	0.68 (0.32–1.44)
Domestic violence (Yes)	–	0.66 (0.37–1.16)	–	0.72 (0.39–1.34)	–	0.60 (0.28–1.11)
Depression (Yes)	–	–	0.95 (0.51–1.77)	–	0.67 (0.34–1.32)	0.67 (0.33–1.33)
Anxiety (Yes)	–	–	0.66 (0.33–1.35)	–	0.83 (0.39–1.80)	0.85 (0.4–1.85)
Suicidal Ideation (Yes)	–	–	1.54 (0.77–3.07)	–	1.28 (0.59–2.77)	1.32 (0.60–2.87)
Self-destructive behavior (Yes)	–	–	**3.17 (1.63–6.26)**	–	**4.28 (2.01–9.33)**	**4.35 (2.02–9.59)**
ADHD (Yes)	–	–	0.57 (0.29–1.04)	–	0.81 (0.36–1.77)	0.83 (0.37–1.85)
Attachment disorder (Yes)	–	–	0.83 (0.45–1.50)	–	0.74 (0.37–1.47)	0.84 (0.41–1.74)
Aggressive Behavior (Yes)	–	–	**4.42 (2.42–8.26)**	–	**5.26 (2.66–10.63)**	**5.75 (2.87–11.84)**
McFadden *R^2^*	0.13	0.01	0.15	0.14	0.28	0.29
Nagelkerke *R^2^*	0.17	0.01	0.19	0.18	0.34	0.35

^1^Contrat jeune majeur (Young Adult Contract: This refers to a type of legal agreement or program that provides support and assistance to young individuals who are transitioning from the status of minors to adults in the child protection context).

Bold font indicates statistical significance (*p* < 0.05).

### Association with child maltreatment

Our results suggest a lack of association between distinct forms of maltreatment and SUD (Table 2). In Model 2, the associations were as follows: [1.43; CI (0.79–2.6); *p* = 0.241] for instances of physical violence, [1.41; CI (0.73–2.59); *p* = 0.29] for instances of sexual abuse, [0.96 (0.54–1.74); *p* = 0.895] for instances of neglect, [0.81; CI (0.44–1.50); *p* = 0.503] for instances of psychological violence, and [0.66; CI (0.37–1.16); *p* = 0.154] for instances of domestic violence. All models confirm the lack of significance obtained in Model 2.

## Discussion

This study examined the association between SUD and forms of child maltreatment, but also with psychological symptoms. To our knowledge, this is the first French study to address this issue in a population of children in child protection care. The main hypothesis of our study was that in the specific population of minors in child protection care, SUD by these minors would be associated with different forms of maltreatment. In our results, on a population of subjects who had suffered abuse and were accompanied by child protection services, the type of abuse did not seem to influence SUD. However, our findings predominantly indicate that SUD is significantly linked to higher age, disengagement from schooling, Self-destructive behavior, and Aggressive Behavior. Moreover, certain models have indicated a significant inverse association between individuals with disabilities and SUD. Children who engage in frequent interactions with their parents, as opposed to those with permanent interactions, exhibit a significant association with SUD.

These results on the absence of association between maltreatment and SUD seem to contradict the existing literature that highlights the association between childhood maltreatment and the risk of substance use in adolescence or adulthood (7, 28). However, most studies compared maltreated children with non-abused children. In our study, all the minors were exposed to childhood maltreatment. We note high odd ratios with physical abuse and sexual abuse. The lack of significance may be an effect of the small size of our consumer group. This could also mean that the type of adversity does not influence SUD, but that it would be child maltreatment in a general sense. The literature points out (29) that child maltreatment can be a stressful life event, often chronic and long-lasting, and where substance use can be used to alleviate the affect (29, 30).

In contrast, our study highlighted strong associations with psychological symptoms, including suicidal ideation. These findings are consistent with previous literature (31, 32). This is important as recent research has noted that children in child protection settings had less suicidal ideation than the general population but were more likely to act on it (33). This aspect raises inquiries into the mentalization capacity of the youth. Previous studies have underscored a correlation between attachment disorders, mentalization, and suicidal ideation (34). Youth in child protection are more exposed to attachment disorders, potentially influencing their mentalization processes and exacerbating the risk of transitioning from ideation to action (35). Further studies should delve into this highly significant aspect to enhance the management of children in child protection.

Regarding psychological symptoms, our results indicate also an association with a risk of aggressive behavior. These findings align with the work of Dória et al. (20) and call for early intervention to prevent progression toward legal action (11). Previous studies have underscored, in the general population, that substance consumption during adolescence is linked to a range of adverse outcomes, including aggressive behavior, antisocial conduct, and school disengagement (36). Substance Use Disorder (SUD) associated with aggressive behaviors acts as predictors of delinquent involvement (23). These youths exhibiting aggressive behaviors tend to identify themselves within peer groups, facilitated by their disengagement from schooling (37). Within these groups, aggressive behaviors and SUD are glorified, thereby escalating the risk of behavioral issues and SUD over the course of years (37). If our study does not measure the bidirectional aspects between SUD and psychological symptoms, it highlights numerous associations that would require further investigation into these dimensions. Indeed, bidirectionality is a complex and significant issue in public health. However, it is not necessarily straightforward. Previous studies had emphasized the bidirectional aspect between SUD and aggressive behaviors (38). On the other hand, a more recent study pointed out a reciprocal association between alcohol consumption and internalized symptoms in an adolescent population (13). However, when the analysis accounted for gender and externalized symptoms, no bidirectional association was found. Despite these contradictory results, the bidirectional relationship between SUD and psychological symptoms has been documented in the general population (12, 39–41), further studies should further investigate populations exposed to adversity, especially in child protection settings.

Beyond the aspects related to symptomatology, our study highlights a significant association between school dropout and substance use. Studies have noted the effects of substance use, particularly cannabis, on school results (42, 43) and on school absenteeism. However, these studies were in general populations. It has been pointed out that children followed by the child protective services were significantly more likely to drop out of school than other children (44), but beyond the effects of maltreatment on school dropout (45), we have little information on the profile of minors. Our results suggest that children who are abused and use substances are more likely to drop out of school. This element must be taken into account in the holistic support of the child. In particular, by strengthening interaction between schools and child protection services, and by making reports to reduce the risk of children dropping out of school.

We propose not a distance but a multi-professional line of action, as it can be developed in the open dialog (46) protocols for network to address the issue of transference, which is partly undermined by the presence of attachment disorders. In this sense, it seems preferable to us to favor institutional psychotherapeutic approaches that favor the presence of a strong, multi-transferential collective (47). This approach would make it possible to fight against the process of desocialisation encouraged by attachment disorders and which can generate the risk of dropping out of school (48).

### Limitations

Our study has several limitations. The main bias of the study is its design. A longitudinal study with repeated measurements to assess placed children would have allowed for greater data reliability. Secondly, the small sample size of the SUD may influence the significance of the results. Thirdly, there may be a recall bias in the case of retrospective data, especially with regard to sexual abuse where it is well known that there may be traumatic dissociation and lower reporting. Finally, the measurement of consumption was not carried out using a scale but from the elements recorded in the files. These are mainly related to a psychological assessment, but may also be the transcript of the observations of the referral educator.

### Conclusion

This study highlights the association between psychological symptoms and susceptibility to psychoactive substance use. Importantly, it highlights a pronounced vulnerability to suicidal ideation and underscores a significant inclination toward aggressive behavior. Moreover, our results emphasize that among the cohort of minors under the purview of child protection services, those who partake in substance use are at an elevated risk of discontinuing their education. These comprehensive findings contribute crucial insights to the nuanced decision-making landscape within this institution (49).

There’s no doubt that exposure to childhood trauma has a profound impact on mental well-being. As evidenced by prior research, it serves as a pivotal determinant that can shape the trajectory of an individual’s psychological health. Addressing the effects of childhood maltreatment and ensuring effective management strategies emerge as paramount endeavors for safeguarding mental wellbeing or positive mental health and prevent the risk of SUD.

## Data availability statement

The datasets used and/or analyzed in this study are available from the corresponding author upon reasonable request.

## Ethics statement

Ethical approval was not required for the study involving humans in accordance with the local legislation and institutional requirements. Written informed consent to participate in this study was not required from the participants or the participants’ legal guardians/next of kin in accordance with the national legislation and the institutional requirements.

## Author contributions

AE and MM co-wrote the first draft of the article. AE, MM, AA, FW, JM, MR, and GS participated in the data collection and correction of the article. GS supervised the work. All authors contributed to the article and approved the submitted version.
